# Geographical mortality distribution of cardiovascular diseases: First report from South Khorasan, Iran

**Published:** 2017

**Authors:** Toba Kazemi, Gholamreza Sharifzadeh, Nahid Borna

**Affiliations:** 1 Birjand Cardiovascular Diseases Research Center, Professor of Cardiology, Birjand University of Medical Sciences, Birjand, Iran.; 2 Birjand Infectious Diseases Research Center, Assistant Professor of Epidemiology, Birjand University of Medical Sciences , Birjand, Iran.; 3 Student Research Committee, Birjand University of Medical Sciences, Birjand, Iran.

In the past decades, cardiovascular diseases were the most common causes of death around the world and in Iran (1, 2). Various factors play an important role in the incidence of CVD; such as environmental, personal as well as life-style.

We studied the geographical distribution of 19142 CVD deaths from 2004 -2010 in South Khorasan. The mean age of the deceased was 59.3±28.9 years. 28.5% deaths were due to CVD. As shown in figure 1, the highest rate of CVD mortality was seen in Sarayan (156.3/100000 population), lowest in Nehbandan (71.2/100000), and in the whole province (122.5/ 100000). One of the known influential factors in the occurrence of CVD is geographical variation; such as urbanism, ruralism, temperature, altitude, and raining (3, 4). South Khorasan is one of the desert wastelands of Iran in which its temperature fluctuates a lot between day and night and this fluctuation may have a role in the incidence of CVD. South Khorasan is one of the poorest regions in Iran. It is believed that the people living in areas with low social-economic status are prone to higher prevalence of cardiac risk factors and influenced by life context (5). Relationship between weather temperature and number of hospitalizations due to CVD is either U or V curve. Weather temperature has a severe role in the incidence of acute heart problems among the two shades of heat and cold (6). With regard to the poverty in South Khorasan, its temperature fluctuation and low rainfall, CVD is expected to be the most prevalent cause of death in the region. CVD mortality is more common in the north and center than in the south of the region. Nehbandan is the southern city of the province. The fewer cases of CVD in Nehbandan might be due to wide distribution of urban and rural spots, few healthcare centers, and short stay of physicians to diagnose the exact cause of death. The effect of geographical direction of a place as in the east, west, north, and south in the incidence of CVD has been examined (7). It has been observed that most European countries such as Germany and France, CVD deaths were more prevalent in the eastern and north-eastern and less in the western and south-western parts of the countries (7). It is necessary for health authorities to study potentially risky spot regions, personal and environmental factors. Development of justice in health requires paying attention to poor regions and making regional decisions regarding specific conditions of every geographical region.

**Figure 1:**
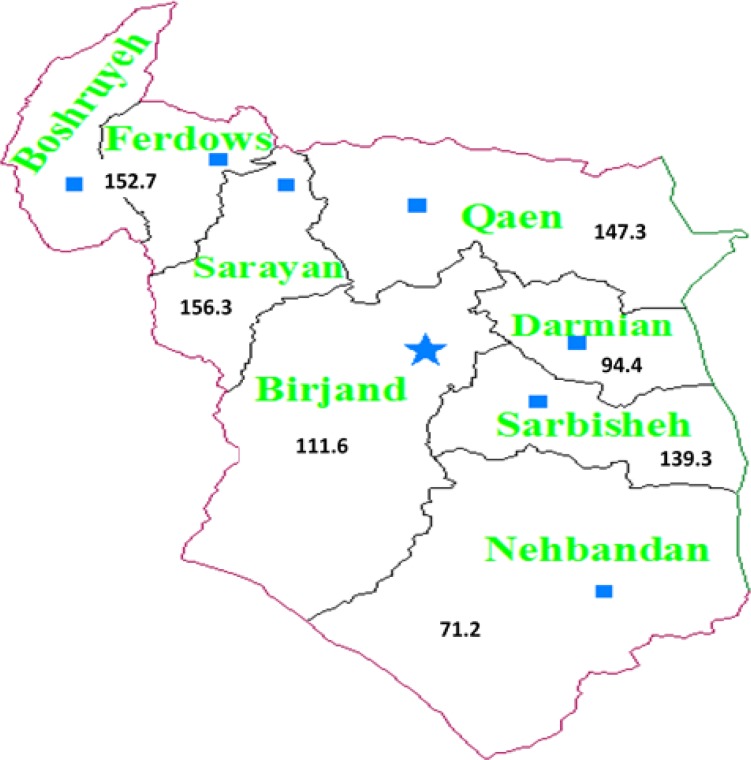
Indirect standard mortality ratio due to CVD in the cities of Southern-Khorasan province (per 100000 populations

## Funding:

The present study was the MD thesis of Nahid Borna with project code 566.
